# Successful Endoscopic Injection Sclerotherapy of High-Risk Gastroesophageal Varices in a Cirrhotic Patient with Hemophilia A

**DOI:** 10.1155/2010/518260

**Published:** 2010-04-29

**Authors:** Kohei Fukumoto, Hideyuki Konishi, Koichi Soga, Ki-ichiro Miyawaki, Hitoshi Okano, Masahito Minami, Naoki Wakabayashi, Shoji Mitsufuji, Norimasa Yoshida, Tomohisa Takagi, Nobuaki Yagi, Yuji Naito, Keisho Kataoka, Toshikazu Yoshikawa

**Affiliations:** Molecular Gastroenterology and Hepatology, Graduate School of Medical Science, Kyoto Prefectural University of Medicine, Kawaramachi-Hirokoji, Kamigyo-ku, Kyoto 602-8566, Japan

## Abstract

A 68-year-old man with hemophilia A and liver cirrhosis caused by hepatitis C virus was referred to our hospital to receive prophylactic endoscopic treatment for gastroesophageal varices (GOV). He had large, tense, and winding esophageal varices (EV) with cherry red spots extending down to lesser curve, predicting the likelihood of bleeding. Esophageal endoscopic injection sclerotherapy (EIS) was performed with a total 15 mL of 5% ethanolamine oleate with iopamidol (EOI). Radiographic imaging during EIS demonstrated that 5% EOI reached the afferent vein of the varices. He was administered sufficient factor VIII concentrate before and after EIS to prevent massive bleeding from the varices. Seven days after EIS, upper gastrointestinal endoscopy (UGIE) showed that the varices were eradicated almost completely. Eighteen months after EIS, the varices continued to diminish. We report a successful case of safe and effective EIS for GOV in a high-risk cirrhotic patient with hemophilia A.

## 1. Introduction

Portal hypertension is one of the major complications of liver cirrhosis and results in the development of esophagogastric varices (EGV). Before virus-free coagulation factor concentrates became available in 1987, most hemophilic patients treated by the concentrates were infected by hepatitis B (HBV) and/or hepatitis C virus (HCV). While these viral infections are frequently asymptomatic at an early stage, liver dysfunction gradually progresses and ultimately leads to liver cirrhosis [1]. In cirrhotic patients with hemophilia, bleeding from EGV leads to a very serious clinical condition, which is difficult to control due to coagulation disorders and decreased platelet count. Therefore, the management of EGV is especially important in these patients in order to improve their prognosis.

We performed prophylactic endoscopic injection sclerotherapy (EIS) with supplementation of factor VIII concentrate for high-risk gastroesophageal varices (GOV) (large, tense, and winding esophageal varices (EV) with cherry red spots extending down to lesser curve) in a 68-year-old Japanese cirrhotic patient with hemophilia A. A single EIS session eradicated the varices almost completely and no recurrence of varix was observed until eighteen months after the treatment, which indicated the usefulness and efficacy of the procedure.

## 2. Case Report

**Figure 1:**
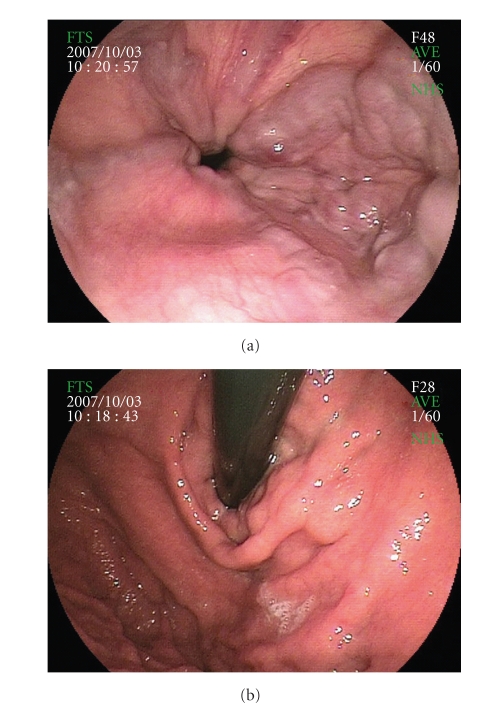
GOV before EIS. (a, b) UGIE showed large, tense, and winding EV with cherry red spots extending down to lesser curve.

 A 68-year-old Japanese man with hemophilia A and liver cirrhosis was referred to our hospital because of large, tense, and winding EV with cherry red spots extending from middle esophagus down to lesser curve, classified as type 1 GOV (GOV1) [2, 3] (Figures 1(a) and 1(b)). He was diagnosed with hemophilia A and chronic hepatitis C at 40 years of age. Hepatitis C was treated with ursodeoxycholic acid (UDCA) and stronger neo-minophagen C (SNMC), while hemophilia A required no medical treatment. The patient had no previous history of bleeding from EGV. On admission, his consciousness level was alert and hepatic encephalopathy was not present. Computed tomography (CT) and ultrasonography (US) showed cirrhotic liver without ascites. Dynamic CT showed that the varices were supplied by the left gastric vein (LGV). Laboratory findings showed the following: alanine aminotransferase (AST), 58 IU/L (12–35 IU/L); aspartate aminotransferase (ALT), 52 IU/L (6–33 IU/L); total bilirubin (T-Bil), 1.08 mg/dL (0.2–1.0 mg/dL); albumin (ALB), 3.5 g/dL (3.9–5.2 g/dL); white blood cell count (WBC), 2600/mm^3^ (3400.2–7300/mm^3^); hemoglobin (Hb), 12.8 g/dL (12.5–15.9 g/dL); platelet count (PLT), 42000/mm^3^(160000–327000/mm^3^); prothrombin time (PT), 86%; factor VIII activity, 16.4% (78.0–165.0%); activated partial thromboplastin time (APTT), 60.4 second (24.2–34.1 second); and Child-Pugh classification, grade A. He was positive for antibody to HCV, but negative for HBV surface antigen and antibody to human immunodeficiency virus (HIV). 

**Figure 2:**
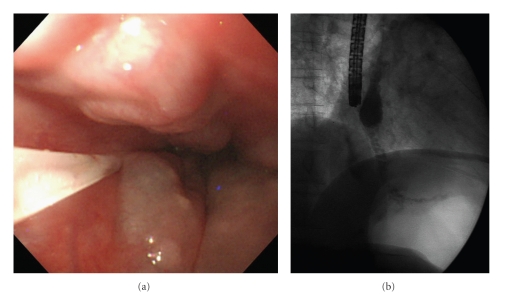
EIS procedure. (a) EIS was performed by intravariceal injection of 15 mL of 5% EOI in total. (b) Radiographic imaging showed that 5% EOI reached the cardiac plexus and LGV.

**Figure 3:**
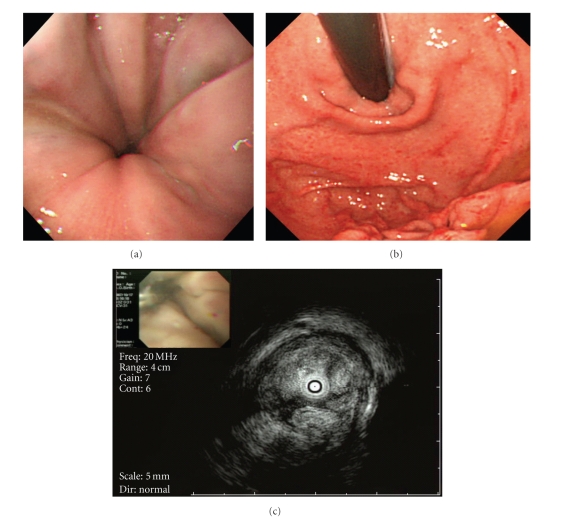
GOV seven days after EIS. (a, b) UGIE showed that the varices were eradicated almost completely without cherry red spots. (c) EUS showed high echoic areas in the lumen of the varices, indicating that the varices were completely thrombosed after EIS.

**Figure 4:**
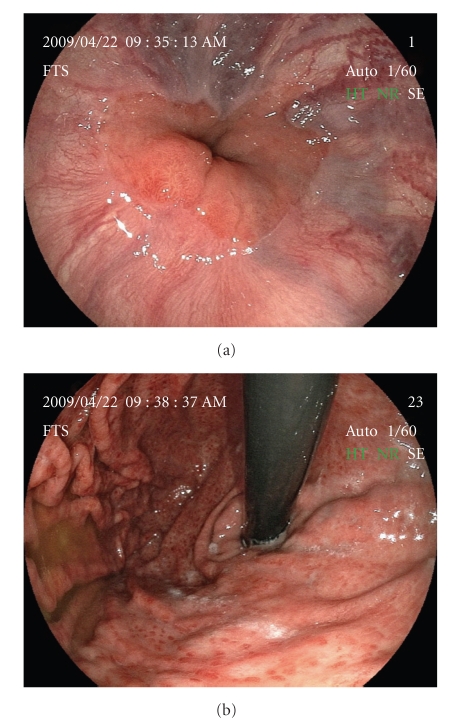
GOV eighteen months after EIS. (a, b) Follow-up endoscopy showed that the varices continued to shrink.

 We obtained written informed consent for EIS and factor VIII concentrate supplementation from the patient before the procedure. According to the guidelines for the management of hemophilia published by the World Federation of Hemophilia in 2005 [4], the patient was administered a dose of 50 U/kg of factor VIII complex supplementation in order to achieve activity greater than 100% of the normal level to prevent massive bleeding from the varices; 3000 U of factor VIII was injected 1 hour before and 12 hours after EIS on the first day, and every 24 hours until the fifth post-operative day, resulting in 104% increase in plasma level of factor VIII activity in the patient after the injections. The total amount of factor VIII supplementation was 21,000 U. Esophageal EIS was performed by intravariceal injection with a total 15 mL of 5% ethanolamine oleate with iopamidol (EOI) for three varices at middle esophagus (Figure 2(a)). Radiographic imaging during EIS demonstrated that 5% EOI reached the LGV, which was the afferent vein of the GOV (Figure 2(b)). No para-operative complications were observed during and after EIS. Seven days after EIS, upper gastrointestinal endoscopy (UGIE) showed that the varices were eradicated almost completely without cherry red spots (Figures 3(a) and 3(b)), and endoscopic ultrasonography (EUS) showed high echoic areas in the lumen of the varices (Figure 3(c)), indicating the formation of thrombi in the afferent vessels as well as the varices. At follow-up endoscopy eighteen months after the treatment, the varices continued to diminish without visible cherry red spots (Figures 4(a) and 4(b)), however, the appearance of esophageal telangiectasia and portal hypertensive gastropathy was observed.

## 3. Discussion

It is very difficult to control hemorrhagic incidents in hemophiliacs because of their coagulation disorders that invasive treatments such as tooth extractions and surgery proved problematic in the past. In recent years, however, as a result of advancements in coagulation factor replacement therapy, hemophiliacs have been able to receive invasive treatments as safely as other nonhemophiliacs. Even invasive endoscopic treatments like sclerotherapy are possible for hemophiliacs through a sufficient supplementation of coagulation factors. The transfusion requirement can be calculated based on the desired increase in activity level targeted to each clinical situation [5]. Generally, the prophylactic endoscopic treatment for EV indicates EIS and EVL. The results of randomized controlled studies evaluating prophylactic EIS have been controversial [6–10]; some studies have shown a significant benefit [6–8], but others have not [9, 10]. The trials including patients with high-risk esophageal varices have illustrated that prophylactic EIS reduces the incidence of the first variceal bleeding and prolongs survival [6, 7]. Gotoh et al. concluded that recurrence of varices was more frequent in patients treated with EVL than EIS, suggesting that EVL was not recommended for prophylactic therapy of EV in liver cirrhosis [8]. In other trials, however, EVL has been shown to be equal in effect to EIS, but with fewer complications [11–13]. 

In the cases of hemophilia with EV, there were, thus far, two reported cases of endoscopic treatment for EV [14, 15], but there was no reported case for GOV in literature. One of the cases was successfully treated by EVL [14]. In this case, the author recommended EVL but not EIS, because EVL produced shallow circular ulcers that resolved more rapidly than the deep linear ulcers caused by EIS [14]. The other patient was treated by EIS [15]. The author concluded that patients could be safely treated with either EVL or EIS if performed by an expert [15]. In these cases, the coagulation factor replacement therapy was performed, and it was deemed as the effective procedure to prevent hemorrhagic incidents after the treatments. In contrast to these cases of EV, however, our patient had gastric varices (GV) in combination with EV. Esophageal EIS also can obliterate associated GOV in a large proportion of patients, depending on the GV type [2, 3] and led to the disappearance of 30% to 60% of GOV1 within six months [2, 3, 16]. This is likely to be caused by caudal flow of sclerosant toward the GV [3]. Because of the possibility that GOV may disappear after esophageal EIS, it has been recommended that in patients with GOV, the EV should first be treated, and if after six months the GV persist, then specific therapy for GV should be considered if indicated [2, 3]. For these reasons, we selected not EVL but EIS and succeeded in eradicating GV almost completely together with EV. Eighteen months after the procedure, the varices continued to diminish.

In the reported case of EVL for EV in a hemophiliac patient, bleeding from one of the ulcers caused by EVL was suspected as the cause of melena eight days after EVL [14]. In addition, one year after two EVL sessions, variceal growth was observed and two additional EVL sessions was needed to achieve variceal eradication [14]. We estimated that the late risk of hemorrhaging from treatment-related ulcers after EIS could be reduced by sufficient supplementation of the coagulation factor concentrates and appropriate intravariceal injection of 5% EOI. Additionally, compared to EVL, the sessions of treatment can be reduced by performing EIS with sufficient occlusion of the afferent vessels. The reduction of the sessions of treatment can also contribute to reducing the expense of coagulation factor supplementation. Because sclerotherapy is considered to be a major surgical procedure for which current guidelines recommend that an activity of 70–100% should be achieved [4], we administered factor VIII complex to the patient to achieve a 100% activity level of factor VIII. The half-life of factor VIII products is about 8 to 10 hours, and thus factor VIII was supplemented every 12 hours on the first day. With sufficient factor VIII supplementation, we performed EIS by stabbing three varices and injecting 5% EOI with time lag adequate to completely occlude patients' afferent vessels, LGV. The supplementation of factor VIII prevented bleeding from treatment-related ulcers after EIS. In this way, a single EIS session eradicated the varices almost completely without any complications. In a follow-up endoscopic study eighteen months after EIS, no recurrent EGV were observed. There has been no other reported case that, like our case, succeeded in eradicating GV together with EV in one session with relatively fewer doses of coagulation factors and without recurrence of varix for a relatively long period. 

In conclusion, when performing endoscopic treatments for high-risk patients such as hemophiliacs, it is very important to appropriately manage their respective conditions through sufficient coagulation factor supplementation in addition to attentively performing EIS in order to prevent hemorrhagic complications.
